# THERapy–Related InterACTion (THER-I-ACT) in Rehabilitation—Instrument Development and Inter-Rater Reliability

**DOI:** 10.3389/fneur.2021.716953

**Published:** 2021-08-06

**Authors:** Thomas Platz, Jonathan Seidel, Andreas Müller, Carolin Goldmann, Ann Louise Pedersen

**Affiliations:** ^1^Neurorehabilitation Research Group, University Medical Centre, Greifswald, Germany; ^2^BDH-Klinik Greifswald, Institute for Neurorehabilitation and Evidence-Based Practice, “An-Institut, ” University of Greifswald, Greifswald, Germany

**Keywords:** assessment, therapy, interaction, rehabilitation, behavior, alliance, feedback, rating

## Abstract

**Objective:** To develop an instrument for the observation of therapeutic communication interactions during rehabilitation sessions and test its inter-rater reliability.

**Methods:** The new instrument THER-I-ACT (THERapy–related Inter-ACTion) has been designed to assess both the frequency and timing of therapeutic interactions in the thematic fields information provision, feedback, other motivational interaction, and bonding. For this inter-rater reliability study, a sample of stroke survivors received arm rehabilitation as either arm ability training, arm basis training, or mirror therapy, or neglect training as individually indicated. Therapy sessions were video-recorded (one for each participant) and therapeutic interactions rated by two independent raters using THER-I-ACT.

**Results:** With regard to the instrument's comprehensiveness to document therapeutic interactions with pre-defined categories the data from 29 sessions suggested almost complete coverage. Inter-rater reliability was very high both for individual categories of therapeutic interaction (frequency and time used for interaction) (intraclass correlation coefficient, ICC 0.91–1.00) and summary scores for the thematic fields of interaction (again for frequency and time used for interaction) (ICC 0.98–1.00).

The inter-rater reliability for rating engagement and being focussed for both the therapist and patient was substantial (ICC 0.71 and 0.86).

**Conclusions:** The observational study documented that by use of the newly designed THER-I-ACT various types of therapy-related communication interactions performed by therapists can be assessed with a very high inter-rater reliability. In addition, the thematic fields and categories of therapeutic interaction as defined by the instrument comprehensively covered the type of interaction that occurred in the therapeutic sessions observed.

## Introduction

Stroke is the second frequent cause of acquired disability (1). Aside from spontaneous recovery, structured multidisciplinary rehabilitation reduces disability and improves functional outcome considerably (2). Such improvements are caused by a bundle of individualized targeted rehabilitation interventions. Such interventions create a clinically relevant benefit if they are specific, i.e., focus on a body function and/or activities to be improved and of high enough intensity (3). In case of recovery from brain damage they have to be tailored to specifically enhance functional recovery by training-induced cerebral reorganization (4).

Aside from the biological mechanism of action any training-related rehabilitation intervention needs to be mediated in the context of a professional relationship between the treating physician or therapist and the patient.

Thus far, rehabilitation research has been addressing effects of specific contents and/or dosages of rehabilitation treatment, while little systematic knowledge about therapeutic interaction during treatment sessions is available.

This is true even though provision of information and establishing social connections are key elements in rehabilitation medicine (5).

It is evident that patients need to be taught and supervised when learning how to perform a prescribed training, i.e., by receiving information about the link between her/his rehabilitation goal(s) and the prescribed training, its elements, mechanism of action, as well as specific procedural knowledge about the training tasks. During the training, patients can further benefit from extrinsic feedback guiding their training behavior, e.g., their approach to the training tasks and their effort (6). Such feedback can be provided as knowledge of performance, KP, i.e., feedback on the nature of the movement pattern used for task accomplishment (e.g., selective movements in a single joint or simultaneous movements in several joints), or knowledge of result, KR, i.e., feedback about the results of a behavior (e.g., time needed to complete a task, precision achieved).

In addition to such information provision and feedback, establishing social connections in therapeutic practice involves “bonding,” i.e., activities that support a positive relationship at a personal level. Acknowledging and showing interest in the person treated, being responsive to her/his individual needs and communication initiatives, actively engaging in conflict solving during therapeutic session, and at time introducing own personal content by a healthcare professional are all aspects of such “bonding.” Indeed, patients in rehabilitation value aspects of a positive personal interaction (7).

Edward Bordin conceptualized “working alliance” as arising from achieving consensus and producing collaboration between therapist and patient in three areas, i.e., 1. “goals” of therapy, 2. the means by which these goals will be achieved (“tasks”), and 3. personal attachments which he labeled “bonds” (8). He developed the concept of work alliance to characterize aspects of relationships and interactions between therapists and patients in psychotherapy, but meanwhile his concept has more widely been adapted in clinical therapeutic research (9). As a consequence, instruments had been developed to assess the subjective quality of work alliance aspects. One such tool is the work alliance inventory, WAI (10).

While work alliance research has received interest in psychotherapy research and beyond, such research has more frequently focused on the retrospective (overall) subjective perception of work alliance, e.g., after a series of therapeutic sessions. The concept has, however, not been used to analyse interactions within therapeutic sessions.

The research reported here set forth to develop an assessment tool for the observation of therapeutic communication interactions during rehabilitation sessions in a theory-driven way and to do so reliably and comprehensively.

The new instrument THER-I-ACT (THERapy–related Inter-ACTion) has been designed to assess both the occurrence/frequency and timing of therapeutic interactions in the domains (thematic fields) information provision, feedback, other motivational interaction, and bonding with further pre-defined categories in each domain (compare Table 1; more details are given in the THER-I-ACT manual provided as Supplementary Material).

**Table 1:** THER-I-ACT: observed thematic fields and aspects (categories).

**Thematic fields (1., 2., 3. …) and individual categories (a., b., c. …)**
1. Provision of information
a. Treatment goal
b. Training specifications
c. Instructions
2. Feedback
a. Knowledge of Performance, KP (unless corrective)
b. KP with positive social stimuli
c. KP with negative social stimuli
d. Corrective KP (cKP)
e. cKP with positive social stimuli
f. cKP with negative social stimuli
g. Knowledge of Result, KR
h. KR with positive social stimuli
i. KR with negative social stimuli
3. Motivational interactions
a. other than KP or KR
4. Bond
a. Showing interest in person treated
b. Personal aspects (treating person)
c. Responsivity
d. Conflict solving
5. Other type of interaction
**Overall ratings**
6. Presence (concentration) and engagement (treating person) (0–10)
7. Focussed attention and engagement (patient) (0–10)

The behavior rated by THER-I-ACT is that of a rehabilitation therapist interacting with a patient during a therapeutic session. Rating is meant to be performed by a rater with clinical experience in the field.

## Methods

### THER-I-ACT (THERapy–related Inter-ACTion): Instrument Design and Piloting Its Use

#### Instrument Design

THER-I-ACT focusses on verbal and non-verbal therapy-related communication by a therapist to the patient being treated.

This communication is documented for different thematic fields and pre-specified aspects within these fields in terms of both the frequency of such communication aspects, the time allocated for it during a therapeutic session, and can be amended by a verbal description of the communication interaction.

Themes and rated aspects of THER-I-ACT are presented in Table 1. For more detailed information see the THER-I-ACT manual in the Supplementary Data.

For each aspect it is documented how many times the communication aspect was observed during the training session (i.e., its frequency), and combining all these instances how much time overall had been allocated to this communication aspect during the training session (i.e., time used for interaction).

In addition to rating these individual aspects, summary scores for both frequency and time used for interaction are generated across individual aspects belonging to a thematic field.

A verbal description of interaction behavior can be documented for both verbal and non-verbal communication by a therapist. In case standardized verbal communication as documented in writing (e.g., standard operation procedures, SOP for the therapy applied) was used, such communication does not need to be described verbally for THER-I-ACT while its occurrence (frequency and timing) is documented.

Therapeutic interactions between a therapist and a patient are not always of communicative nature e.g., therapists might observe a patient or might support a patient physically; such interactions are not documented with THER-I-ACT.

In addition to the thematic fields and categories as mentioned above, the instrument documents an overall rating to what degree the therapist is considered present, concentrated and engaged in the therapeutic situation, and as how focussed on and engaged in the training tasks a patient is perceived during a training session.

#### Piloting the Instrument

After the test items and a manual had been established (TP) piloting of their use was performed. For that purpose, video-recorded therapeutic sessions of 13 stroke survivors receiving arm rehabilitation or neglect therapy were rated by two independent raters (AM, JS). The piloting demonstrated the feasibility to use THER-I-ACT's to document occurrence and timing of therapeutic interaction and indicated its comprehensiveness (i.e., covering the therapeutic interactions well). Piloting also indicated areas where aspects and the manual needed to be edited to support unambiguous and hence reliable rating of observed behavior. For example it is important to discriminate episodes of therapeutic interaction before their characteristics (i.e., thematic field and category they belong to and their length) can be determined. In general, the trigger for interaction and the overall communication intention and frame (including its temporal extension) are identified first; the thematic field and category of interaction are to be chosen next.

After piloting and improving the manual accordingly an inter-rater reliability study was conducted.

This research report presents inter-rater reliability data for the quantitative aspects frequency and time allocated for therapeutic interaction (both for individual categories and summary scores for thematic fields) as well as the overall rating of the therapist's presence and engagement and of the patient's focussed attention and engagement during rehabilitation sessions.

### Reliability Study

#### Patient Population and Therapy Applied

Stroke survivors of the regional community who could benefit from arm rehabilitation (having functional deficits caused by arm paresis) or visuospatial neglect therapy (suffering from visual neglect) were informed by newspaper advertisement and information leaflets in outpatient services about the offer to participate in the observational study that included individualized therapy (free of charge).

Participants were offered a week of intensified outpatient rehabilitation with 5 therapeutic sessions, each lasting appr. 1 h. Type of training offered depended on the individual needs and included the arm ability training for patients with mild arm paresis (11), the arm basis training (12), or mirror therapy (13) for stroke survivors with severe arm paresis, and neglect therapy including optokinetic stimulation, saccade training, and visual exploration (14) for patients with visuospatial neglect. Therapy was provided by 4 staff members.

Stroke survivors with an interest to participate in the study were screened for eligibility criteria (stroke leading to arm paresis or neglect) and were included if they were eligible and gave informed consent. Prior to recruitment the study was approved by the institutional review board (IRB) (i.e., ethics committee, EC).

Baseline assessment that also served as basis for individual treatment decisions included sociodemographic information, information regarding the type of stroke and time post stroke, a standardized neurological examination (NIH Stroke Scale, NIHSS) (15), assessment of disability (Barthel Index) (16), emotional distress (Hospital Anxiety and Depression Scale, HADS) (17), and the assessment of the functional deficits to be treated, i.e., selective movement capacity (Fugl-Meyer, arm motor score) (18) (patients with moderate to severe arm paresis), manual and finger dexterity (Box and Block Test, BBT and Nine Hole Peg Test, NHPT) (19, 20) (patients with mild arm paresis), and spatial orientation of visual attention (Neglect Test, NET) (21) (patients with visuospatial neglect).

#### Video Recording and Rating

When a training commences the necessity to provide information and to establish a professional interpersonal relationship is highest. Hence, a variety of information provision interaction, feedback, and bond supporting behavior can be expected in such sessions. Therefore, video recordings of the first therapeutic session with each participant were used for the inter-rater reliability study.

Video recording was performed in such a way that the video-based observation of facial expressions and gestures of both the therapist and patient was facilitated. For that purpose, a large mirror (on wheels) was placed at an angle of 45 degrees to the camera axis next to therapist and patient so that aspects perpendicular to the camera's axis could be captured. By this set-up a single video recording provided two perpendicular perspectives simultaneously.

Two raters (CO, AP), an occupational therapist and a physical medicine and rehabilitation specialist that were not involved in the THER-I-ACT piloting received a rater training with introduction into the manual's contents and its application using a few examples of video recordings of therapeutic sessions. Once the two trained raters could apply the manual in a valid way (as assessed by rating of individual video-recordings) both raters independently rated the video-recorded therapeutic session of all participants of the reliability study using THER-I-ACT.

#### Sample Size Determination

The study was designed to test the inter-rater reliability of the THER-I-ACT (both for individual categories and summary scores) when applied by two independent raters.

For clinical purposes, an at least moderate inter-rater reliability as indicated by an intraclass correlation coefficient, ICC of 0.60 or higher was warranted. For testing H_0_: ICC = 0.20 (lack of reliability) vs. H1: ICC = 0.60 (moderate reliability) with two independent raters and alpha = 0.05 and beta = 0.20, a sample of 27 participants would be necessary (22). A sample of that magnitude was planned to be recruited so that documented ICCs of 0.6 or higher could be regarded as substantiated.

### Statistical analyses

Baseline characteristics of the study population are presented using descriptive statistics (counts, mean, standard deviation).

For all qualitative outcome measures, i.e., frequencies and time used for individual categories of interaction, their summary score for thematic fields, and overall ratings of presence and engagement by the therapist and of focussed attention and engagement by the patient the following statistics were calculated:

Mean and standard deviation (sd) for each rater (rater 1, R1, and rater 2, R2) and intraclass correlation coefficient, ICC.

The ICC is the appropriate statistic to assess the consistency of ratings for interval and ratio levels of measurement (23). In the presented research, two-way random effects models have been used for ICC estimation since each item was assessed by both raters. Specifically, ICC (1, 2) according to Shrout and Fleiss (24) had been calculated using a SAS macro written by Robert M. Hamer, Ph.D., Virginia Commonwealth University, 2-7-1991.

## Results

The study population consisted of stroke survivors with a broad age and fair sex distribution, that were well-balanced with regard to side of brain affected, had mild to moderate disability (Barthel Index), no to moderate level of emotional distress (Hospital Anxiety and Depression Scale, HADS), and were characterized by a broad range of time post stroke (from weeks to many years) (compare Table 2).

**Table 2:** Study population characteristics and type of training (*n* = 29).

	**Mean/sd**	**Min–max**	***n***
	**n (%)**	**n (%)**	
Age (mean/sd, min–max)	63.2/9.2	49–80	
Sex (female, male) [n (%)]	11 (38%)	18 (62%)	
Stroke type (ischemic, ICH) [n (%)]	26 (90%)	3 (10%)	
Affected brain (left, right) [n (%)]	13 (45%)	16 (55%)	
Time post stroke (weeks) (mean/sd, min-max)	181/273	8–1,158	
NIHSS (0–42) (mean/sd, min-max)	5.0/3.8	1–14	
Barthel Index (0–100) (mean/sd, min-max)	86/17	45–100	
HADS (0–42) (mean/sd, min-max; n)	12.7/7.3	2–27	28
Treated syndrome (paresis^1, 2, 3^ neglect^4^)	25 (86%)	4 (14%)	
Type of therapy (AAT^1^, ABT^2^, MT^3^, NT^4^)	15, 6, 4, 4		
FM Arm^2, 3^ (0–66) (mean/sd, min–max; n)	21.3/15.0	4–49	10
BBT^1^ (blocks/minute) (mean/sd, min–max; n)	38.3/11.3	17–58	15
NHPT^1^ (sec) (mean/sd, min–max; n)	61.8/38.9	25.3–147	15
NET^4^ (0–170) (mean/sd, min–max; n)	112.5/31.3	67.5–140	4

*AAT, Arm Ability Training; ABT, Arm Basis Training; BBT, Box and Block Test; FM Arm, Fugl-Meyer Arm Motor score; HADS, Hospital Anxiety and Depression Scale; ICH, intracerbral hemorrhage; NET, Neglect Test; NHPT, Nine Hole Peg Test; NIHSS, National Institute of Health Stroke Scale; NT, Neglect Therapy; min, minimum; max, maximum; MT, Mirror Therapy; sd, standard deviation.*

*Superscript numbers (AAT^1^, ABT^2^, MT^3^, NT^4^) indicate the different types of therapy and how they relate to both the treated syndromes and the tests used for baseline assessment, respectively*.

Within each category of clinical presentation (i.e., mild arm paresis, moderate to severe arm paresis, and neglect) that could be treated with one type of therapy offered the respective assessment scores for body functions or activities again indicated a substantial range of severity (compare the range of scores for the FM Arm, BBT, NHPT, and NET, resp.; Table 2).

All therapies offered were prescribed and applied with a higher relative frequency for the arm ability training as it was more frequently individually indicated in the study sample.

Taken together the study sample represented a relatively broad spectrum of stroke survivors, different therapies were prescribed and observed.

With regard to therapeutic interaction the ratings by the two independent raters had been very consistent while considerably varying across subjects and across categories (compare Tables 3, 4).

**Table 3:** THER-I-ACT observations and inter-rater-reliability–individual categories (*n* = 29).

**Thematic field and individual category**	**Frequency of interaction**			**Time used for interaction (in sec)**		
	**R1 (mean/sd)**	**R2 (mean/sd)**	**ICC**	**R1 (mean/sd)**	**R2 (mean/sd)**	**ICC**
1. Provision of information						
a. Treatment goal	1.4/0.6	1.4/0.6	1.00	131/93.5	131/94.4	1.00
b. Training specifications	1.2/0.8	1.2/0.8	1.00	72.6/56.7	73.3/57.3	1.00
c. Instructions	248/186	251 /188	1.00	1208/487	1203/484	1.00
2. Feedback						
a. Knowledge of Performance, KP (unless corrective)	26.6/44.7	27.7/46.9	1.00	39.1/59.6	40.4/60.7	1.00
b. KP with positive social stimuli	24/38.3	23.2/36.5	1.00	38.3/58.8	37.1/55.8	1.00
c. KP with negative social stimuli	0.0/0.0	0.03/0.2	0.00	0.0/0.0	0.03/0.2	0.00
d. Corrective KP (cKP)	1.1/1.9	0.8/1.4	0.91	2.7/7.0	2.3/6.8	0.99
e. cKP with positive social stimuli	0.0/0.0	0.0/0.0	–	0.0/0.0	0.0/0.0	–
f. cKP with negative social stimuli	0.0/0.0	0.0/0.0	–	0.0/0.0	0.0/0.0	–
g. Knowledge of Result, KR	85 /105.7	84.9/107.2	1.00	173.9/163.4	174.2/167.2	1.00
h. KR with positive social stimuli	31.5/28.4	31.0/28.5	1.00	72.6/70.0	69.7/67.4	1.00
i. KR with negative social stimuli	0.0/0.0	0.0/0.0	–	0.0/0.0	0.0/0.0	–
3. Motivational interactions						
a. other than KP or KR	1.9/2.4	2.2/2.6	0.98	4.3/6.5	4.9/7.1	0.98
4. Bond						
a. Showing interest in person treated	21.1/16.2	21.7/16.3	0.99	55.0/35.4	56.5/35.1	0.99
b. Personal aspects (treating person)	0.4/0.8	0.4/0.8	1.00	5.9/12.5	5.7/11.8	1.00
c. Responsivity	26.7/30.7	28.1/32.8	1.00	100.6/100.6	99.6/105.5	1.00
d. Conflict solving	0.3/0.6	0.3/0.7	0.96	4.8/11.5	4.5/11.1	1.00
5. Other type of interaction	1.1/1.2	1.1/1.2	0.99	6.8/9.0	6.7/8.7	0.99
6. Presence (concentration) and engagement (treating person)						
(0–10)	8.9/1.5	8.7/1.0	0.71			
7. Focussed attention and engagement (patient)						
(0–10)	8.7/1.5	8.6/1.3	0.86			

*ICC, intra class correlation coefficient; KP, knowledge of performance; KR, knowledge of result*.

Both for individual categories of therapeutic interaction in terms of frequency of occurrence and time used for interaction (ICC 0.91–1.00) (Table 3), and for summary scores across categories within thematic fields (ICC 0.98–1.00) (Table 4) consistency between two independent raters was almost perfect. An exception was the type of feedback “knowledge of performance, KP” combined with negative social stimuli where ratings were inconsistent (ICC 0.00), yet such interactions were almost absent during the video-recorded sessions leading to the lack of consistency.

**Table 4:** THER-I-ACT observations and inter-rater-reliability–summary data (*n* = 29).

**Themes (summary for individual aspects)**	**Frequency of interaction**			**Time used for interaction (in sec)**		
	**R1 (mean/sd)**	**R2 (mean/sd)**	**ICC**	**R1 (mean/sd)**	**R2 (mean/sd)**	**ICC**
1. Provision of information	251/186	254/188	1.00	1,412/570	1,407/569	1.00
2. Feedback	168/107	168/109	1.00	326/169	324/170	1.00
3. Motivational interactions other than KP or KR	1.9/2.4	2.2/2.6	0.98	4.3/6.5	4.9/7.1	0.98
4. Bond	49/43	50/46	1.00	166/136	166/135	1.00
5. Other type of interaction	1.1/1.2	1.1/1.2	0.99	6.8/9.0	6.7/8.7	0.99
Duration of therapeutic session (in min)	71/19	71/19	1.00			

*ICC, intra class correlation coefficient; KP, knowledge of performance; KR, knowledge of result*.

The ratings of the therapist's presence and engagement and of the patient's focussed attention and engagement during a rehabilitation session were somewhat less consistent between raters (ICC 0.71 and 0.86, resp.), but still substantial.

Aside from addressing the psychometric property of inter-rater reliability for THER-I-ACT the data also provides insight into the type and “distribution” of therapeutic interaction observed when the above-mentioned therapies were applied with stroke survivors.

Treatment goal-related interaction and training specifications occurred more or less once per session as a longer communication episode.

Instructions were on average very frequently given, yet of much shorter length (time allocated per episode on average).

Feedback was given both as knowledge of performance, KP and knowledge of results, KR. Both were provided with short episodes, presented in a neutral mode or associated with positive social stimuli. Feedback associated with “negative social stimuli” was hardly ever observed.

With regard to interactions promoting inter-personal bond the categories “showing interest in person treated” and “responsivity” occurred relatively frequently while therapist seldom shared own “personal aspects” and hardly had to engage in “conflict solving.”

The two categories that intended to capture other, not specifically pre-defined categories, i.e., the category “motivational interactions other than knowledge of performance, KP or knowledge of results, KR” and the thematic field “Other type of interaction” indicated almost none such (not otherwise categorized) interaction.

Both the therapists being rated and the participating patients were rated as showing a high degree of engagement and focussed attention during the therapeutic sessions.

## Discussion

While therapeutic interaction is considered a key element in rehabilitation therapy (5), to date little systematic research has been performed to address the topic in more detail.

For one, therapeutic interaction has more frequently been a focus of research in psychotherapy. There, concepts had been developed that could be used for other forms of therapy as well. Indeed, work alliance has systematically been measured in clinical therapeutic research related to psychotherapy and beyond (9).

Second, work alliance research addressed the overall (retrospective) impression of work alliance as rated by either therapists or patients. So far, no instrument has been available that intends to measure within session therapeutic interaction as performed verbally or non-verbally by therapists both self-initiated and as response to a patient's communication trigger directly and comprehensively.

The new instrument THER-I-ACT (THERapy–related Inter-ACTion) has specifically been designed for that purpose, i.e., to document therapy-related communication interactions performed by therapists during rehabilitation therapy. It respects the important work alliance dimensions “goal,” “task,” and “bond” (9) and further specifies types of extrinsic feedback (6). With that theoretical background and specific knowledge from rehabilitation therapy development (4, 11, 12) a comprehensive detailed manual-based instrument was developed (TP). It measures both the occurrence/frequency and timing of therapeutic interactions in the domains “information provision,” “feedback,” and “bonding” with a variety of pre-defined categories in each thematic field (compare Table 1).

After piloting the test this reliability study had been conducted; its methods and results are reported following the Guidelines for Reporting Reliability and Agreement Studies (GRRAS) (25).

By using a non-selective sample of stroke survivors with different clinical presentations, mild to moderate disability and varying therapeutic needs and hence different therapeutic approaches being applied video-recorded therapeutic sessions with a relevant scope and variability of therapeutic interactions could be used for observation.

In that way, the newly developed instrument THER-I-ACT could be tested based on ecologically valid clinical user scenarios. Thereby, the instrument's capability to capture therapeutic interactions in various therapeutic situations reliably and comprehensively could be assessed quantitatively.

With regard to the test's comprehensiveness to document most if not all therapeutic interactions with pre-defined categories the data suggested almost complete coverage. Categories that were to be used for “any other interaction” had been rarely observed.

On the other side, some pre-defined categories of therapeutic interaction were also infrequently observed in the study sample. This does not speak against their relevance for capturing therapeutic interactions in general, because such interaction might well occur in other situations and would be very important to note (e.g., feedback being associated with negative social stimuli or active problem-solving interaction).

All other categories were more frequently observed with considerable variability of time used for interaction across these categories. Provision of information, feedback, and bond-supporting interactions were all documented for the therapeutic sessions analyzed.

Overall, a multitude of pre-defined aspects of therapeutic interaction was observed with variable expression across therapy sessions, and rather big differences in the time used for different types of interactions.

The independently performed rating by the two raters nevertheless showed a very high consistency both for individual categories of therapeutic interactions (frequency and time used for interaction) (ICC 0.91–1.00; Table 3) and summary scores for the thematic fields of interaction (again for frequency and time used for interaction) (ICC 0.98–1.00; Table 4).

The rating of engagement and being focussed for both the therapist and patient was somewhat less consistent, while the inter-rater reliability of these ratings was still substantial (ICC 0.71 and 0.86, resp.; Table 3).

This very high inter-rater reliability for the observation of therapeutic interaction was achieved by two rehabilitation professionals who had received prior training in the application of the THER-I-ACT instrument. While this does not imply that such a high inter-rater reliability would uniformly be observed in other situation, it documents the possibility that appropriately trained health care professionals can discriminate and document diverse types of therapeutic interaction reliably. This observation is reassuring since human-human interactions are complex.

Caution must, however, equally be expressed. Appropriate rater training is required before the instrument can be applied in a reliable and valid way. The delineation of communication episodes, recognizing the communication intention and the temporal frame associated with it all need to be comprehended and necessitate both a very good understanding of the instrument (as described in the manual) and clinical experience in the field.

The ability to document so diverse therapeutic interactions as defined by THER-I-ACT reliably is of great value for clinical therapy research.

By use of the instrument spontaneous therapeutic behavior and its variation with different type of therapies and patient characteristics, its relevance for subjectively perceived work alliance by patients, and for the magnitude of therapeutic benefit achieved can all systematically be evaluated.

Such knowledge could be fundamental for education for healthcare professionals.

In addition, intervention studies could address the effects of a systematic variation of therapeutic interaction provided during therapeutic sessions. It is well-conceivable that not only content and dosage of training therapy is generating a differential therapeutic benefit (e.g. 11; 19), but also the way therapists interact with their patients. Provision of information, e.g., why and how a training supports the attainment of personalized treatment goals, or the appropriate type of feedback for a given training, maybe best associated with positive social connotations, might well have an independent or modifying effect on therapeutic outcome. Furthermore, patients with emotional distress might benefit from more “showing interest in the other person” leading to a reduction of emotional distress and improved resources for training.

A further application of such therapeutic interaction knowledge, is the use of humanoid therapists in therapy, e.g., socially interactive robots that supervise training as therapeutic assistants (e.g., www.ebrain-science.de). Such digital implementations could greatly benefit from more refined systematic knowledge about therapeutic interactions. If their social therapeutic interaction could resemble human therapeutic interaction behavior including its individualization, their therapeutic assistance might considerably be enhanced and more acceptable for patients.

In summary, this reliability study documented that by use of the newly designed THER-I-ACT (THERapy–related Inter-ACTion) various types of therapy-related communication interactions performed by therapists during rehabilitation therapy can be assessed with a very high inter-rater reliability. In addition, the thematic fields and categories of therapeutic interaction as defined by the instrument comprehensively covered the type of interaction that occurred in the therapeutic sessions observed. The possibility to reliably document complex types of therapeutic interactions opens a window of opportunity for clinical therapeutic research both with a focus to systematically assess therapeutic interaction behavior and understand reasons for its variation, for interventions studies with a focus on therapeutic interaction, and for their implementation with humanoid social robots to be used as therapeutic assistants.

## Data Availability Statement

The datasets presented in this article are not readily available because secondary to personal data protection regulations the data can only be used by the research group and for the purposes declared when obtaining written informed consent. Requests to access the datasets should be directed to Thomas Platz, thomas.platz@uni-greifswald.de.

## Ethics Statement

The studies involving human participants were reviewed and approved by the Ethikkommission an der Medizinischen Fakultät der Universität Greifswald. The patients/participants provided their written informed consent to participate in this study.

## Author Contributions

TP designed and wrote the manuscript. JS, AM, CG, and AP critically reviewed the manuscript for intellectual content. All authors contributed to the article and approved the submitted version.

## Conflict of Interest

The authors declare that the research was conducted in the absence of any commercial or financial relationships that could be construed as a potential conflict of interest.

## Publisher's Note

All claims expressed in this article are solely those of the authors and do not necessarily represent those of their affiliated organizations, or those of the publisher, the editors and the reviewers. Any product that may be evaluated in this article, or claim that may be made by its manufacturer, is not guaranteed or endorsed by the publisher.
