# Supplementary material for: THERapy–Related InterACTion (THER-I-ACT) in Rehabilitation—Instrument Development and Inter-Rater Reliability
Source: Front Neurol. 2021 Aug 6;12:716953. doi: 10.3389/fneur.2021.716953 (PMC8377230; doi:10.3389/fneur.2021.716953)
Supplement: Supplementary file 1 [file Data_Sheet_1.PDF]

THER-I-ACT (THERapy–related InterACTion)

Manual

Objective

Objective of THER-I-ACT is to document therapy-related communication interactions performed by therapists during rehabilitation sessions.

General description of THER-I-ACT

THER-I-ACT focusses on verbal and non-verbal communication by therapists.

This communication is documented for different thematic fields and pre-specified aspects within these fields in terms of both the frequency of such communication aspects, the time allocated during a therapeutic session, and the verbal description of the communication interaction by therapists.

For each aspect it is documented how many times the communication aspect was observed during the therapeutic session and combining all these instances how much time overall had been allocated to this communication aspect during the therapeutic session.

The verbal description addresses both verbal and nonverbal communication by therapists.

Interactions between a therapist and a patient are not always of communicative nature. E.g. therapists might observe a patient or might support a patient physically; such interactions are not documented with THER-I-ACT.

In this manual communication interaction is meant when the term therapy-related interaction is used. For any such therapy-related interaction the specific type of interaction is determined. Details that characterise such aspects are given below.

Therapy-related interactions can be very short, e.g. a brief comment “yes” when a target was correctly hit during a reaching and aiming task, or “lengthy”, e.g. when training specifications are explained for a set of training tasks on a first training day. What matters for the discrimination of single therapy-related interaction events is the frame of their content. In case of feedback given for hitting a single target the content frame is the result of whether or not the target was hit during a discrete aimed movement and hence such interaction is completed with and restricted to a single word feedback (“yes”). Training specifications for a set of training tasks refer to a much wider frame of contents and consequently complex explanations will be necessary to complete that specific therapy-related interaction (all belonging to that interaction event).

Thematic fields and aspects to be assessed

The following thematic fields and their respective aspects (categories) are to be assessed for training sessions when using THER-I-ACT.

For each aspect (category) it is documented how many times the communication aspect was observed during the training session (i.e. its frequency) and combining all these instances how much time overall had been allocated to this communication aspect during the training session (i.e. time used for interaction).

In addition to rating these individual aspects, summary scores for both frequency and time used for interaction are generated across individual aspects (categories) belonging to a thematic field, i.e. summary scores for frequency and timing of therapeutic interactions in that thematic field.

The documentation can be amended by a verbal description of the communication interaction.

Thematic field "Information provision"

"Information provision" (other than feedback) includes the aspects goal-related communication, training specifications and training instructions given by a therapists as described below.

There might be instances when a patient her- or himself responds to treatment goal-related communication, training specifications, or training instructions given by a therapists and consequently the therapist might again react to this patient behaviour in turn within the overall explanatory communication. Such "back and forth" interactions are considered part of the primary interactions (e.g. treatment goal-related communication, or provision of training specifications or training instructions). Such complex interactions are considered a single communication event. Timing of such therapeutic interaction events is restricted to the parts when the therapist is the active communication partner.

Aspect "Treatment goal":

Rehabilitation therapy is goal-oriented. Any communication that is related to information about or agreement on treatment goals is documented in this category. In rehabilitation, goal-orientation includes the reflection of the current status (based on diagnostics/assessment and/or patient-reports), the status aimed for, and hence the gap that should be bridged by rehabilitation as well as the therapeutic approach (decided on) to achieve the therapeutic goal. The general link between current and desired status and any intervention planned to support the achievement of goals make all of these part of goal-oriented communication. More detailed information about the therapy itself is, however, coded separately as "training specifications".

Aspect "Training specifications":

The focus here are the characteristics of the training. The information given is how the training can be described.

A specific rehabilitation treatment can be characterised by its contents and structure, e.g. what are the training tasks, how are they structured, why do they support the achievement of treatment goals. Any knowledge about its mechanism of action or evidence about benefit, harm or acceptability to patients. Providing any information related to these training specifications is documented under this category.

Aspect "Procedural knowledge and training instructions":

Before a training starts or during a training session a therapist might provide specific and explicit procedural knowledge and give instructions to a patient about how to perform training tasks. Such communication is documented under the category "procedural knowledge and training instructions". When such information is provided in the form of explicit procedural knowledge not using a "command" structure, the intention to provide knowledge on how to perform training tasks is a pre-requisite for this category; in addition, any explicit instruction is part of this interaction aspect.

Such information might be very brief or longer in nature. When explicit procedural knowledge on how to perform training tasks and instructions involve multi-step actions that are introduced or instructed together this counts as a single (longer instruction). When instructions do, however, occur as brief commands for single discrete movements or actions and occur sequentially, but separately for each discrete movement or action, then each brief instruction is considered an interaction event and hence is counted separately (and documented with its short duration).

Explicit procedural knowledge on how to perform training tasks and instructions can specify how training tasks are to be performed using one´s body, i.e. by body functions. Such instructions address the "performance" aspects related to a training task. Or else, such knowledge and instructions can address how a training task is to be performed to meet aspects in an outside world perspective. Such instructions address "result" aspects related to a training task.

Example: For aimed movements a therapist might instruct a patient to keep the trunk stable while reaching with the arm forward (performance aspect, i.e. how to perform the task using one´s body), or to always hit the target with each aimed movement ("result"-related instruction / outside world perspective).

Instructions can occur before or during a training task. Feedback is given after training behaviour, or when training task behaviour has started and is "under way" (for further information see thematic field "feedback" below). Instructions given during or after a training task can be distinguished from feedback by their "command" character (instruction) as compared to the "knowledge provision" character of feedback.

The distinction can be difficult when the instructions are corrective in nature, implying an observed (past) or suspected (present or forthcoming) unwanted performance or result. Such an instruction can be classified as "corrective instruction" while it does not qualify for the aspect "feedback". Feedback provides information ("knowledge"), but does not itself imply a command, while instructions always ask for or request a behaviour. In the case of corrective instructions such instructions do imply information that warrant a correction of training behaviour (or results) while they are not "pure" feedback (information provision).

Example: "Please, don't move your trunk while making these reaching movements" implies observed (past) or suspected (present or forthcoming) unwanted performance, i.e. trunk movement, while it is an (corrective) instruction and not feedback. Feedback would be: "I saw that you moved your trunk during the last reaching movement".

Corrective instructions may at time be given non-verbally, e.g. with a gesture or a corrective intervention by the therapist. Such non-verbal behaviour qualifies as instruction, if it has a component that demands an adaptation from the patient being treated.

Thematic field "Feedback"

Feedback provides information about and / or comments on current or completed behaviour. It can address the way an activity (or movement) is performed, i.e. by body functions. Such feedback is called "knowledge of performance (KP)". Or else, it can address the result of behaviour as measured from an outside world perspective. This is called "knowledge of results (KR)". Both forms of feedback are characterised by explicit information provided about training behaviour (KP) or its results (KR).

Example: An aimed movement might be performed with a jittery ataxic movement that leads to missing the target. Feedback regarding the way the movement had been performed (jittery), is KP, while feedback as to the precision at the target (target missed; distance from target) is KR.

Such "objective" feedback as either KP or KR can be either given indicating a status (current behaviour or result) or a change (compared to previous trials). In addition, compared to the training goal such an objective status or change can be either positive or negative in nature. Any improvement would be positive, any deterioration negative.

In the instance of KP feedback that provides an objective account of a negative status or change of behaviour is termed "corrective". Such feedback is given to guide a patient to change behaviour from an insufficient and/or maladaptive behaviour while performing the training tasks to a different more adaptive way to perform the training tasks (while not being expressed as instruction).

Example: Patient is asked to perform a selective flexion movement at her or his wrist without flexor activities at other joints of the paretic arm. The patient does, however, flex both wrist and elbow. The therapist provides corrective (objective) KP when indicating that she or he flexed the elbow while flexing the wrist.

For KR, any objective status or change that is negative in nature is not called “corrective”. Such KR does provide orientation, but does not imply an indication how to correct the behaviour.

So far, “objective” feedback as either KP or KR had been described. At times, therapists combine such feedback (be it positive or negative) with social stimuli that are not “objective”, but rather “judgemental” with a social notion of strengthening or discouraging a behaviour.

Example: “well done” (positive), “I know you could do better” (negative).

Such social stimuli and whether they are positive or negative in expression need to be distinguished from positive or negative objective aspects of feedback. Any combinations are possible.

When feedback is rated one needs to assess first whether KP or KR is given. Next, assess whether the objective feedback given is positive or negative in nature (if KP was negative in nature this would be “corrective” feedback). Finally, assess whether any social stimuli had been used in addition to the feedback given and whether they had been positive or negative in nature.

Aspect “Knowledge of performance” (excluding “corrective knowledge of performance”):

Knowledge of performance gives feedback on how a training task was performed. This might include behavioural strategies, or the quality of movements or aspects of innervation in motor rehabilitation. The conceptual frame for knowledge of performance are body functions. Such body functions include mental planning and the type and sequence of the way the body was used to perform a training task. Knowledge of performance addresses the question how the training task was done using body functions. Such communication is documented under this aspect unless it is “corrective knowledge of performance” or directly combined with either positive or negative social stimuli.

Aspect “Corrective knowledge of performance”:

Knowledge of performance gives feedback on how a training task was performed. KP feedback that provides an account of a negative status or change of behaviour on how a training task is or was performed in terms of body functions used, is termed “corrective”. Such communication is documented under this aspect unless it is directly combined with either positive or negative social stimuli.

Aspect “Knowledge of performance with positive social stimuli”:

Knowledge of performance might be presented in a “neutral” way (facts only) or with additional features that are not information / knowledge themselves, but meant to encourage training behaviour further by positive social stimuli. Such communication is documented together with the knowledge of performance provided (i.e. “knowledge of performance” or “corrective knowledge of performance”).

Example: “Very good, you did amazing.”

Aspect “Knowledge of performance with negative social stimuli”:

Knowledge of performance might be presented in a “neutral” way (facts only) or with additional features that are not information / knowledge themselves, but meant to discourage training behaviour further by negative social stimuli. Such communication is documented together with the knowledge of performance provided (i.e. “knowledge of performance” or “corrective knowledge of performance”).

Example: “Well, I know you could do better.”

Aspects “Corrective knowledge of performance with positive social stimuli” and “Corrective knowledge of performance with negative social stimuli”:

Similarly, corrective KP can be combined with either positive or negative social stimuli and is coded accordingly.

Aspect “Knowledge of result”:

Knowledge of results (KR) provides information about the results, i.e. the external consequences of a patient’s behaviour during training. Such information could be provided as single short comment on success or failure (e.g. “yes” or “not hit” with aimed movements), refined information on results for single actions, or as summary KR on errors, accuracy, timing, or other results of behaviour from an outside world perspective. This type of communication is documented under this aspect unless it is directly combined with either positive or negative social stimuli; if the latter is the case provision of knowledge of result is documented under the corresponding aspect, i.e. either with positive or with negative social stimuli.

Aspect “Knowledge of result with positive social stimuli” and “Knowledge of results with negative social stimuli”:

As with knowledge of performance knowledge of result can be combined with either positive social stimuli to further encourage training behaviour or with negative social stimuli to further discourage training behaviour. Such communication is documented together with the knowledge of result provided.

Thematic field ”Other motivational support”:

Feedback is related to a patient’s behaviour and provides information about the performance or results of behaviour as indicated above. There are situations where motivational support is provided that is, however, not directly linked to knowledge of performance or knowledge of

result. Such motivational support could be provided by verbal and or nonverbal encouragement or discouragement and is documented here.

Thematic field "Work alliance support "

Aspect "Interest for the other person":

This aspect documents behaviour by a therapist that shows an interest in the patient and her or his individual situation that is neither addressed by the more general aspects of treatment goals nor feedback in terms of knowledge of result or knowledge of performance.

Simple forms of this type of interaction can be: "Are you ready?" or "Are you well seated?" or "Do these training tasks cause any pain in your arm?"

Aspect "Introducing own personal content":

This aspect documents interactive behaviour by the therapist where by the therapist provides individual personal information related to her- or himself.

The interaction aspect "Introducing own personal content" may occur separately or can be embedded in any other interaction specified in this manual. When it occurs it is always coded independently of the communication context it is linked to.

In case the interaction aspect "Introducing own personal content" is embedded in another overall communicative frame, the overall communication frame will be coded as a single event (as will be the interaction aspect "Introducing own personal content"), yet the time used for the interaction aspect "Introducing own personal content" will be deduced from the time measured for the overall communication frame.

Example: A therapist explains the training specifications for the training to be commenced. During this explanatory communication the therapist reports about own personal training experiences and thereafter continues with the explanation of the training specifications. For such a communication situation, explanation of training specifications would be counted as single event, as would "Introducing own personal content". Timing for training specifications would be the overall measurement of time used for the communication minus the time used for "Introducing own personal content".

Aspect "Responsivity":

The aspect responsivity documents the interaction situation where a patient shows a verbal or nonverbal behaviour, e.g. a complaint that can be regarded as a communication trigger from the patient, and documents whether and how the therapist responded to this specific behaviour by a patient.

"Responsivity" is not coded when a patient her- or himself responds to treatment goal-related communication, training specification, or training instruction given by a therapists and consequently the therapist reacts to this patient behaviour in turn. Such "back and forth" interactions are considered part of the primary interactions (e.g. treatment goal-related communication, provision of training specifications, or training instructions).

Aspect "Solving conflicts":

During therapy sessions patients are advised to perform and fulfil tasks and to achieve certain goals with these task. Conflicts might arise when e.g. a patient does not want to perform the prescribed tasks, does not do so with an engaged manner, wants to quit, or is unhappy with his or her own performance endangering motivation to continue, or when otherwise bodily complaints arise that question whether the intended activities can be continued. Such and other conflicts may arise during training sessions. The aspect "Solving conflicts" documents the interaction behaviour by a therapist to solve such problems.

Thematic field "Other"

There might be situations when the aspects provided above do not cover all aspects of communication interaction by therapists during training sessions. If they are considered relevant they are be documented under the umbrella term "other" communication aspects.

Rating scales

While the above mentions thematic field and aspects are meant to code interaction events, the following two thematic fields are meant to provide an overall (subjective) rating for both the therapist and the patient for a therapeutic session.

Thematic field "Presence (concentration) and engagement":

This Likert rating scale documents a (subjective) rating (whether and) to what degree the therapist is considered present, concentrated and engaged in the therapeutic situation. It is an overall rating for individual training sessions observed. The rating ranges from "0, not present, concentrated and engaged at all" to "10, very high degree of presence (concentration) and engagement" for a therapist during the training session.

Note of caution: The rating should not be mistaken for a rating of "quality of therapy". It only addresses to what degree the observer had the impression that a therapist was present, concentrated and engaged in the therapeutic situation. E.g., there might be situations when a patient does not welcome a very high degree of presence (concentration) and engagement

by a therapist since she or he might perceive a lack of “freedom” or “choices” on her or his side by very close attention. In addition, “quality of therapy” is influence by various factors not addressed here. The rating of ”presence (concentration) and engagement” should be performed independently of any consideration of an overall “quality” rating for the therapeutic session, but merely focus on the rater’s perception of the therapist’s ”presence (concentration) and engagement”.

Thematic field “Focussed behaviour”

Rehabilitative training, especially when performed with a restaurative intention, e.g. the intention to restore an altered brain function, needs networks within the central nervous system (CNS) to re-organise, to functionally adapt, and hence to improve their ability and efficiency to be clinically successful. If training-based, in many if not all instances the achievement of such a goal would rest on repetitive training schedules that are adapted to the level of performance of the individual (at the individual performance limit) and focus on the target domain(s) of interest (e.g. brain function to be improved). As such a training will likely achieve a functional benefit, if the patient is focussed on and engaged in the training tasks throughout the training session. The more the patient is focussed on and engaged in the training tasks, the more the training tasks address the target domain(s) of interest (e.g. brain function to be enhanced) and enable the patient to train at her or his performance limit in that domain(s), the more likely or bigger the training effect might be. This thematic field documents the rater’s subjective impression how focussed on and engaged in the training tasks a patient was perceived during the training session observed.

Accordingly, this Likert rating scale documents a (subjective) rating (whether and) to what degree the patient is considered focussed on and engaged in the training tasks during the training session. It is an overall rating for an individual training session observed. The rating ranges from “0, not focussed on and engaged in the training tasks at all” to “10, very high degree of being focussed on and engaged in the training tasks” for a patient during the training session.

Such rating needs to be performed against the background knowledge considering a patient’s clinical syndrome. Some aspects of a patient’s neurological syndrome may, if not adequately taken into account, cause an incorrect assessment.

Example: A patient with Parkinson’s syndrome might suffer from a reduced capacity for mimic expressions and a paucity of (spontaneous) movements. Such a clinical presentation might lead to an erroneous judgement of a “passive attitude” and consequently the assumption of a low degree of focused and engaged behaviour and hence an invalid low score. It is therefore mandatory that any neurological and other conditions that might act as possible “confounders” for the assessment of a patient and the degree of being focussed on and engaged in the training tasks are taken into account.

Note on verbal description of communicative behaviour

For each thematic field and the pre-specified aspects a verbal description of both nonverbal and verbal communicative behaviour can documented.

There are standards for communication for certain neurorehabilitation therapies. In case standardised verbal communication as documented in writing was used, such communication does not need to be described verbally for THER-I-ACT while its occurrence (frequency and timing) is documented.

In case a behaviour by a patient triggers a communicative interaction by therapist, such behaviour is also described (compare aspects "Responsivity" and "Solving conflicts").

The verbal description is accompanied by a descriptor "v" for verbal communication and "nv" for nonverbal communication and by a "T" for activity by therapist and "P" for behaviour by the treated patient.

Considering the rating scales, verbal explanations for ratings can be added.

Greifswald, 29.05.2021 Prof. Dr. med. Thomas Platz [1,2]

[1] Neurorehabilitation research group, University Medical Centre, Greifswald, Germany, and

[2] BDH-Klinik Greifswald, Institute for Neurorehabilitation and Evidence-Based Practice, "An-Institut," University of Greifswald, Greifswald, Germany

t.platz@uni-greifswald.de
